# Impact of Crisis Communication Strategies on People’s Attitudes toward Behavioral Guidelines Regarding COVID-19 and on Their Trust in Local Officials

**DOI:** 10.1007/s13753-022-00424-3

**Published:** 2022-08-01

**Authors:** Gerrit Hirschfeld, Meinald T. Thielsch

**Affiliations:** 1 Faculty of Business, CareTech OWL – Center for Health, Welfare and Technology, University of Applied Sciences Bielefeld, 33619 Bielefeld, Germany; 2 Department of Psychology, University of Münster, 48149 Münster, Germany

**Keywords:** COVID-19, Crisis communication, Germany, Local officials, Situational Crisis Communication Theory

## Abstract

Situational Crisis Communication Theory (SCCT) guides responses of corporations in crises. We tested how COVID-19 related crisis communication strategies affect trust in mayors and the acceptance of behavioral measures. A total of 561 participants (53% female) with a mean age of 50 years took part in an online experiment in which we systematically manipulated the mayor’s crisis communication strategy (deny crisis, diminish role in crisis, rebuild relationship after taking responsibility, bolster reputation, no response) and pre-crisis reputation (good past crisis management, bad past crisis management). Age, gender, and education served as covariates. We also tested the predictive power of personal concern regarding the COVID-19 pandemic, as well as internal and external control convictions. In our preregistered analysis, we found that crisis communication strategies had no significant effect on participants’ ratings of behavioral measures, but they affected participants’ trust in the mayor. The deny crisis strategy resulted in the most unfavorable ratings, while the effects of the other strategies were moderated by the mayor’s pre-crisis reputation. Additional exploratory analyses showed that individual concern and trust were important predictors for the acceptance of behavioral measures. Even though we did not find any effects of communication strategies on behavioral measures, our results support SCCT’s utility in guiding communication strategy during a pandemic.

## Introduction

Effective and widely available vaccines are a key to address the COVID-19 pandemic. However, lack of vaccines, a significant proportion of citizens who refuse to get vaccinated, or the emergence of new variants such as the omicron-variants make it necessary that citizens follow behavioral measures such as keeping a distance from and limiting one’s contact with other people (Ferguson et al. 2020). Accordingly, a large number of studies have tried to identify psychological, social, and demographic variables that are associated with people’s acceptance and adoption of key behavioral measures, such as keeping a distance, wearing face masks, regularly washing hands, and so on (Dohle et al. 2020; van Mulukom 2020; Kukowski et al. 2021; Schneider et al. 2021; Sulik et al. 2021; Šuriņa et al. 2021).

However, while knowledge about such associations is important from a theoretical perspective, it cannot easily be translated into recommendations about the actions political or private actors should take. First, several of the factors that previous studies have explored—for example, gender, party preference, or religiosity—cannot easily be changed. So knowing that women are more likely to wear masks does not lead to a more effective campaign to improve mask-wearing. Second, these studies have mainly used correlational designs, which are very limited in the causal interpretations they afford. However, there is also a growing number of experimental studies that investigate the impact of different forms of messaging on people’s intentions to engage in various behavioral measures to prevent COVID-19 (Bilancini et al. 2020; Capraro and Barcelo 2020; Everett et al. 2020; Jordan et al. 2021). These studies manipulated messages that described different reasons for adhering to behavioral measures—washing hands, keeping a distance, and so on, and measured participants’ intention to adhere to behavioral measures. They found small effects of different types of messages on participants’ intentions.

In the present study, we used a similar experimental paradigm to test the impact of different crisis communications used by mayors when they introduce more stringent behavioral measures against a localized outbreak of COVID-19. We focused on the crisis communications by mayors because, as local officials, they are responsible for implementing governmental policies to protect the public and for overseeing the implementation of local decrees (such as mask mandates or restricting public events). Furthermore, mayors often lead the local crisis management teams responsible for managing the pandemic response (Thielsch et al. 2021). As demonstrated by the actual responses of mayors in Italy (The Guardian 2020) and Germany (Westdeutsche Allgemeine Zeitung 2020), large differences exist in the crisis communication strategies employed. The main medium that mayors can use to communicate with their citizenry is interviews with the media and press releases.

The theoretical background of our study is the Situational Crisis Communication Theory (SCCT) (for a review, see Coombs 2007). The SCCT was developed in the 1990s by Coombs and Holladay (1996) to address questions of corporate responsibility in the face of crises—for example, reports of damaged products or mistreatment of employees. The appeal of the SCCT was to develop guidelines that corporations could follow to minimize damage to their reputation following a crisis. Crises in this framework are any events that pose a potential risk to the reputation of a corporation. Even though this theory was developed in the corporate domain, it has also been used successfully to study how public actors respond in times of crisis, and specifically the COVID-19 pandemic (Coombs 2020). The basic tenet of the SCCT is that the communication strategies of an organization have to match the specific type of crisis that the organization faces. To this end, the SCCT defines different clusters of crisis types that mostly differ in the perceived control an organization has over the crisis. In the victim cluster the organization is also a victim of the crisis, in the accidental cluster the organization shares some responsibility for the crisis, and in the intentional cluster the organization is the main responsible party for the crisis.

Since the decisions taken by mayors with respect to the COVID-19 crisis—for example, to enforce mask mandates—had an effect on the progression of the pandemic, the crisis with relation to mayors can be classified in the accidental cluster. Depending on the crisis cluster, different response strategies are recommended (Coombs 2007). The most important are: (1) denying a crisis exists; (2) diminishing one’s own role in the crisis; (3) rebuilding the relationship after taking responsibility for the crisis; (4) bolstering one’s reputation by reminding others of past positive behavior; and (5) giving no response. The SCCT predicts that for a crisis in the accidental cluster, the deny strategy can only be used successfully when addressing crises for which the organization has no responsibility. The diminishing strategy can be used as successfully as the other strategies if there is a certain amount of goodwill. A large body of empirical research has investigated the direct effect of crisis communication strategies and possible moderating variables (Coombs and Holladay 1996; Coombs 2007; Hegner et al. 2016; Beldad et al. 2018). A positive pre-crisis reputation can buffer the effects of a crisis on the organization’s reputation (Hegner et al. 2014). Since we investigated the reputations of local officials we assumed that a central aspect of these reputations is trust (Mayer et al. 1995).

Trust is indispensable for effective communication between political leaders and citizens (Lewis and Weigert 1985). Bish and Michie (2010) reviewed different predictors of behavioral measures and found that in the context of a pandemic, trust is an important predictor of behaviors. Several recent studies also support the idea that the public’s acceptance of behavioral measures is positively related to trust in politicians and scientists (Dohle et al. 2020; Guglielmi et al. 2020; van Mulukom 2020). However, there are also studies that indicate paradoxical effects of trust, that is, lower levels of adherence in participants with very high levels of trust in the government (Guglielmi et al. 2020; Wong and Jensen 2020). Different aspects of trust need to be teased apart. Here we focused on the general belief that authorities tell the truth, that is, what in the risk domain is called “social trust” (Siegrist 2021) and assumed that trust and trustworthiness are distinct, as they can vary independently (Mayer et al. 1995; Meeßen et al. 2020). For instance, if a person has a generally low disposition toward trusting politicians, they might even distrust a political leader that is highly trustworthy.

The aim of the present study was to test whether crisis communication strategies can be used to influence mayors’ reputations and the public’s acceptance of and intention to engage in behavioral measures. Our hypotheses are based on the SCCT (Coombs 2007) as well as past experimental research into how different forms of leaders’ messaging affect the public (Capraro and Barcelo 2020; Everett et al. 2020). We assumed that (H = Hypothesis) different crisis communication strategies produce differences in people’s responses—their behavioral intentions to engage in (H1a) and accept (H1b) protective behavioral measures; in how they rate the trustworthiness of the mayor (H1c: benevolence; H1d: integrity; H1e: competence); in their trust in the mayor (H1f); and in their intention to vote for the mayor (H1g). We expected that the deny strategy would result in the lowest acceptance of behavioral measures and least trust compared to all other strategies. Our second set of hypotheses (H2a−H2g) is similarly based on the SCCT, specifically on research that shows that a leader’s pre-crisis reputation can have a buffering effect, that is, we expected an interaction between pre-crisis reputation and crisis communication strategy (Hegner et al. 2016; Beldad et al. 2018). Hypotheses (H2a−H2g) state that the differences (H1a−H1g) are moderated by a mayor’s pre-crisis reputation, where a high pre-crisis reputation buffers the effects of the crisis communication—that is, we expected larger differences in H1a−H1g when the mayor’s pre-crisis reputation is low. Our third set of hypotheses was based on studies that show strong associations between trust and acceptance of and adherence to behavioral measures (Dohle et al. 2020; Guglielmi et al. 2020; van Mulukom 2020). Hypothesis H3a and H3b state that differences in acceptance (H3a) and behavioral intentions (H3b) are mediated by overall trust. In addition, we ran two exploratory analyses. The first aimed to test whether weaknesses in our experimental manipulation contributed to the lack of significant effects. The second aimed to investigate whether demographic variables, control beliefs, and personal concerns related to COVID-19 influenced people’s behavioral intentions.

## Methods

In order to investigate these hypothesis, we conducted an online experiment, which used a 5 × 2 (five different crisis communication styles and two levels of pre-crisis reputation) between-subject design. After reading one of several manipulated newspaper stories about the introduction of novel guidelines to stop the spread of COVID-19, the participants rated their intention to adhere to and acceptance of these behavioral guidelines.

### Participants

Participants in the study were recruited through the German online panel PsyWeb.^1^ Participating in this panel is completely voluntarily, and members agree to receive invitations for scientific studies; they can unsubscribe and delete their personal data at any time. Sample size was determined a priori to achieve sufficient (f = 0.25; Power = 95%; Alpha = 5%) sample size for our moderation-hypothesis H2a−H2e, and a minimum of 400 participants had to be included. In order to achieve this sample size the whole panel was invited to participate. The invitation to the study included information on the topic of COVID-19 communication and the length of the questionnaire. As no major changes were made between the pre-test of the questionnaire and the actual study questionnaire, the eight respondents who took the pre-test questionnaire were included in the study. Of the 886 participants who started the questionnaire, 623 completed the full survey. Another 62 participants were excluded from further analysis due to unrealistically short responses in comparison to the response length of all participants, no variance in their responses, or a wrong answer to an item that required participants to select a specific response category to an item to check the participants’ attention—for example, “For quality assurance, please click on the answer ‘senseless’ here.” (Meade and Craig 2012). The final 561 participants were mostly female (53% female) and had a mean age of 50 years. The education level of 65% of the participants was *Abitur* (German university entrance qualification) followed by *Mittlere-Reife* (16%, a general certificate of secondary education) and Fachhochschulreife (13%, German entrance qualification for applied universities). The final dataset, which included 561 participants, is available at https://doi.org/10.5281/zenodo.4889821.

### Manipulation of Crisis Communication Strategy and Pre-Crisis Reputation

Participants read one out of a total of 10 different versions of a newspaper article describing a fictional scenario about a localized surge in COVID-19 cases. The different versions of the newspaper article were modeled after an article describing an outbreak in the German city of Hamm in October 2020. All articles started with the same short introductory paragraph describing a rapid increase in cases in the fictional town of Sonnenfels (the article mentioned 650 cases/100,000 inhabitants a week; the German average infection rate at the time of the study was around 150 cases/100,000 inhabitants a week). After this, the articles described the same three novel behavioral measures that were introduced in the city: People in the town were expected to (1) wear masks in public at all times; (2) register private gatherings containing more than 10 people, as well as cancel/not have private parties with more than 20 people; and (3) not attend Sonnenfels’s annual village fair and the open Sunday,^2^ as the city canceled these events. These behavioral measures were much stricter than the restrictions that were actually in place in Germany in November 2020. After these sections of the article, the two-part manipulation began (see Table 1 for the manipulations of the communication strategy and pre-crisis reputation). We manipulated how the article described the mayor’s specific communication strategy—deny, diminish, rebuild, bolster, no response—and the mayor’s pre-crisis reputation (high versus low). This resulted in 10 different versions of the article. Participants were randomly allocated to the conditions, resulting in sample sizes for the conditions between 52 and 61 (median = 56) participants. The questionnaire was available online from 6 to 30 November 2020, with most respondents (88.04 %) participating in the first week of the survey period. No major shifts in policy regarding behavioral measures or shifts in the federal communication strategy occurred during this time in Germany. Completing the questionnaire on average took about seven to nine minutes (median = 7.46 min; mean = 8.77 min, SD = 6.18).

**Table 1:** Overview of experimental conditions

Condition	Description	Manipulation—Statement by the Mayor
Communication strategy	Deny: Claim that no crisis exists	In my opinion the numbers cannot be used to draw strong conclusions about the risks of the public in Sonnenfels. First, mostly young persons are infected for whom COVID-19 is not dangerous. Second, the rise in numbers can be attributed to increased testing
	Diminish: Play down the city’s responsibility	We as a city only have a limited impact on the infections. Many people, for example those commuting to work in other cities, get infected there but are counted as cases here. It is self-evident that if the numbers go up in neighboring cities, that numbers in Sonnenfels will go up, too
	Rebuild: Take responsibility	As the city administration we are well aware of our responsibility towards the citizens and will do our utmost to counter this worsening of the crisis. We have requested help from other cities, increased our testing capacities and optimized our internal organization
	Bolster: Remind public of past good crisis management	Sonnenfels is a city that cares about the issues of its citizens. We have done a very good job during the first wave and have come through the crisis very well so far, also due to the dedication of the employees of the city administration. But even we as a city administration cannot work in the same quality as before because of sick employees
	No response	The new rules were published on the city’s website. The mayor was not available for further comments.
Pre-crisis reputation	Positive	During the first wave of COVID-19, at the beginning of 2020, all members of the city council and the citizens praised mayor Meißner for his engagement and flawless crisis management
	Negative	During the first wave of COVID-19, at the beginning of 2020, all members of the city council and the citizens criticized mayor Meißner for his lack of engagement and bad crisis management

In the study, the statements of the mayor were presented in German. The table shows a translation.

### Measures

After participants read the description, they were asked to respond to a series of questions with regard to the fictional scenario. For a complete list of items, experimental manipulations, and instructions see https://doi.org/10.5281/zenodo.4889821.

#### Trustworthiness of the Mayor

To measure the trustworthiness of the mayor in the scenario, we used the TrustDiff scale (Brühlmann 2019). TrustDiff is a semantic differential that consists of 10 items that assess three different dimensions of trustworthiness: benevolence (for example, ignoring–caring), integrity (for example, dishonest–honest), and competence (for example, inept–resourceful). Participants used a seven-point scale to indicate for each of the 10 word pairs which of the two descriptors was more fitting for the mayor. The TrustDiff scale has been extensively validated to measure trustworthiness in web settings and has good psychometric properties. Cronbach’s alpha in the present sample was 0.89, 0.93, and 0.93 for the benevolence, integrity, and competence scales, respectively.

#### Trust in the Mayor

To measure trust in the mayor, we provided three statements regarding the fictional mayor (“I can trust the mayor,” “I rely on what the mayor says,” “I have no reservation about relying on what the mayor says”), which had to be answered on a 7-point scale ranging from 1 (does not apply at all) to 7 (applies completely). Cronbach’s alpha in the sample was 0.95. We also asked participants to indicate on a scale from 0 to 100% how likely they would be to vote for this mayor in a fictional upcoming election.

#### Acceptance and Intention Measures

The acceptance and intention measures were based on the questionnaire developed by Dohle and colleagues (2020). Both scales consist of nine items, naming different behavioral measures (keeping a distance from other persons, regularly washing hands with soap, sneezing into the arm, wearing a mask in public, working from home, canceling private meetings with more than 20 persons, registering personal meetings with more than 10 persons, avoiding large gatherings, and avoiding contact with other people). The measures were selected because they were widely discussed as possible next steps in the COVID-19 response at the time of the study. For the acceptance measure, participants were asked to rate the utility of the different behavioral measures on a scale from 1 (utterly useless) to 7 (very useful). For the intention measure, participants were asked to estimate how often they would comply with these behavioral measures on a scale from 1 (never) to 7 (always). Cronbach’s alpha in the sample was 0.81 for the acceptance measure and 0.79 for the intention measure.

#### Control Variables: Perceived Control and Level of Concern

To measure perceived control, we used the IE-4 scale that measures internal and external control convictions (Kovaleva et al. 2014). The scale consists of four items ranging from 1 (does not apply at all) to 7 (applies completely) that measure two types of conviction—internal control convictions (for example, “I am my own boss”) and external control convictions (for example, “Fate often gets in the way of my plans”). We used a single item (“How concerned are you about the current COVID-19 situation?”), to which the participants responded on a scale of 1 (“not at all concerned”) to 6 (“very concerned”). Both variables were included as covariates in the analysis as described below.

#### Attention and Manipulation Check

We used three additional items to check the manipulation and participants’ attention. The manipulation check consisted of two single-choice items. The first asked participants to summarize the statement the mayor gave as part of the newspaper article. Participants could choose one of five different descriptions, each of which described one communication strategy. The second item asked whether the mayor’s past performance was positive or negative. Attention was checked by including one item into the ratings of acceptance of measures that stated “For quality assurance, please click on the answer ‘senseless’ here.” (Meade and Craig 2012).

#### Pre-test

To check for any technical problems—for example errors in the programming of the online questionnaire, typos, or questions that were not easy to understand—a pre-test was performed. For this eight student assistants at the Department of Psychology were sent a link to the final questionnaire and asked to complete the questionnaire and comment on any difficulties they encountered. The participants who completed the pre-test did not report any serious issues.

### Data Analysis

The data analysis was conducted in accordance with the analysis plan that was established and preregistered before data collection began.^3^ Hypotheses H1a−H1e and H2a−H2e were tested using a 5 × 2 MANCOVA (Multivariate Analysis of Covariance)—Dependent Variables (DVs): intention, acceptance, trust-Benevolence, trust-Integrity, trust-Competence; AV1: crisis communication strategy; AV2: pre-crisis reputation; control variables: age, gender, education. Whenever the MANCOVA analysis yielded significant overall results, these were followed-up by using corresponding separate ANCOVAs for the individual DVs. Relevant to the evaluation of H1a−H1g were the main effects of the crisis communication strategy. Post hoc comparisons for the factor communication strategy were performed using Tukey’s HSD Test. Relevant to the evaluation of H2a−H2g was the interaction between crisis communication strategy and pre-crisis reputation. The effect sizes are reported as omega squared (ω) and Cohens’ *f*. Hypothesis H3 was tested using separate mediation analyses, one for acceptance and one for behavioral intentions, each contrasting the no-response strategy to one of the other four response strategies, resulting in eight mediation tests overall. In all analyses, we estimated the average causal mediation effect (ACME) with the mediation package in R (Tingley et al. 2014). The mediator model described mediator trust as a function of crisis communication strategy, level of control, and level of anxiety about the pandemic. The outcome model described the level of acceptance (H3a) and the behavioral intentions (H3b) as a factor of the mediator trust, the crisis communication strategy, and the level of perceived control and anxiety about the pandemic. Conditions 1−4 (deny, diminish, rebuild, and bolster) were individually contrasted to condition 5 (no response). The significance of the ACMEs was assessed using the bca-corrected bootstrap (Tingley et al. 2014).

Additionally, we ran two exploratory analyses. The first used participants’ perceptions of the communication strategy as assessed in the manipulation check instead of the assigned condition. The second used hierarchical regressions to predict behavioral measures using demographic characteristics (step 1), control convictions (step 2), personal concern regarding the COVID-19 pandemic (step 3), and trust (step 4).

The study was approved by the ethics board of the University of Münster’s Faculty 7, Psychology & Sports Science (2020-57-MT). All materials, raw data, and analysis scripts are publicly accessible.^4^

## Results

In the following we report the results of our manipulation check before describing the results of the planned analysis followed by the results of the exploratory analysis.

### Manipulation Check

Overall, the manipulation check indicated that the majority of participants chose the intended descriptor for the deny, rebuild, and no-response categories, but not for the diminish and bolster categories (Table 2). For both these conditions, the majority of participants instead chose rebuild as the best summary. In contrast, the vast majority (81%) stated that they perceived the manipulation about the mayor’s past performance as intended in the study design. Table 3 shows the descriptive statistics for the individual variables as well as their interrelation.

**Table 2:** Responses to the manipulation-check items in the different conditions

Manipulation item	Condition				
	Deny	Diminish	Rebuild	Bolster	No response
Deny	**50**	1	0	1	1
Diminish	5	31	1	4	2
Rebuild	26	**49**	**82**	**57**	39
Bolster	13	16	22	38	11
No response	10	5	11	5	**58**
Do not know	5	5	2	2	6

The highest frequencies per condition are printed in bold.

**Table 3:** Means, standard deviations (SDs), and correlations

Variable	Mean	SD	1	2	3	4	5	6
1. Intention	6.05	0.85						
2. Acceptance	6.01	0.82	0.83**					
3. Benevolence	4.49	1.40	0.17**	0.15**				
4. Integrity	4.78	1.45	0.21**	0.23**	0.81**			
5. Competence	4.47	1.42	0.19**	0.20**	0.85**	0.84**		
6. Trust	4.41	1.66	0.25**	0.26**	0.77**	0.82**	0.83**	
7. Vote	51.07	26.86	0.19**	0.21**	0.70**	0.75**	0.76**	0.81**

*p < 0.05; **p < 0.01

### Planned Analysis

**Fig. 1:**
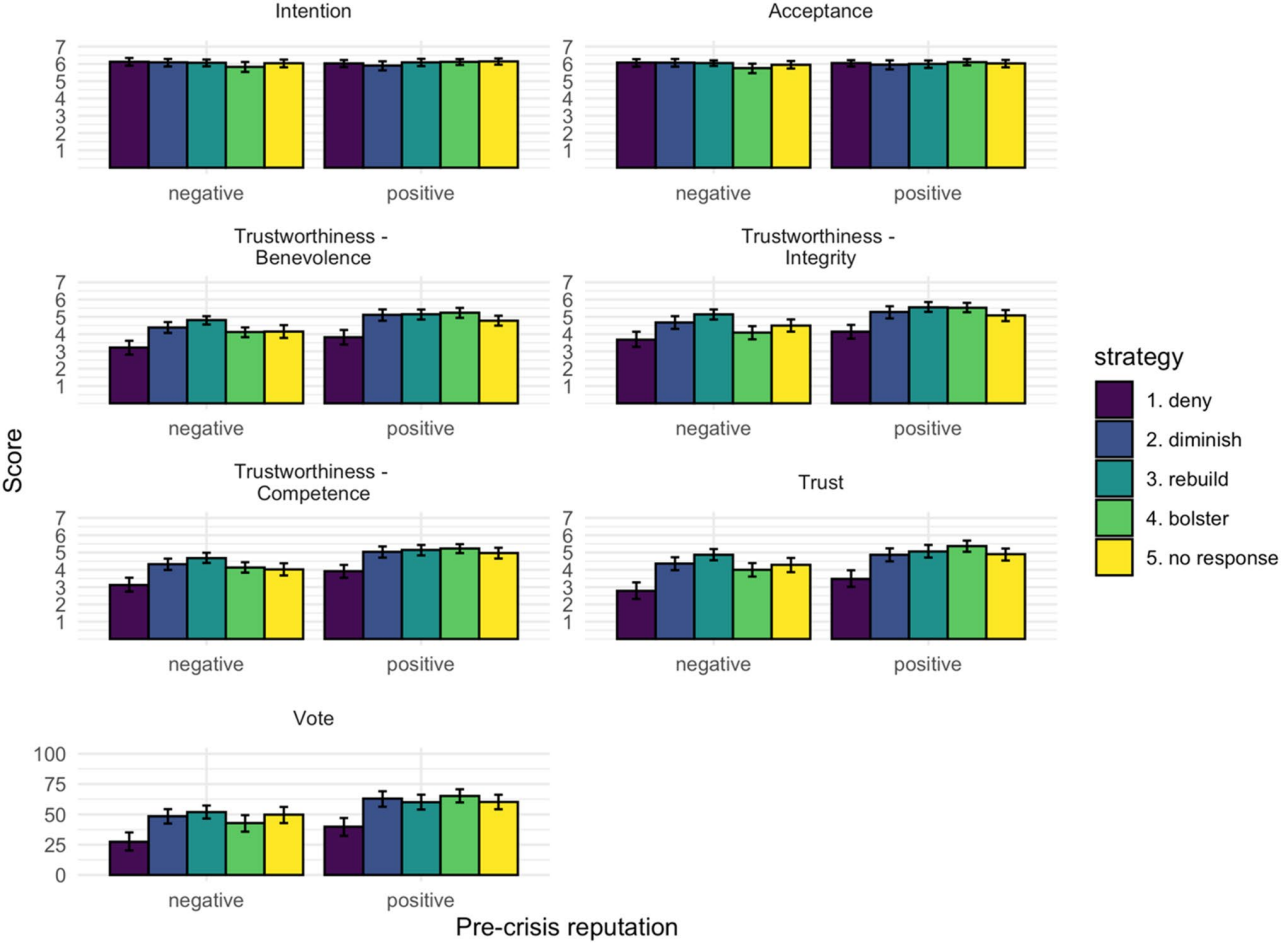
Mean scores for all conditions. Error bars indicate 95% CI (Confidence Interval). Effects of the experimental variables Strategy and Pre-crisis reputation (negative versus positive) and their interaction were found for benevolence, integrity, competence, trust, and intention to vote.

Hypotheses about the main effects of communication strategy (H1) and the buffering effect of pre-crisis reputation (H2) were tested using MANCOVA to compare the mean in the different conditions with regard to the outcome variables (Intention, Acceptance, Benevolence, Integrity, Competence, Trust, Vote; Fig. 1). The overall MANCOVA revealed significant effects for:

- strategy (H1): Pillais’ Trace = 0.23; F(24, 2188) = 5.59; p < 0.001; ω = 0.06; *f* = 0.25;
- pre-crisis reputation: Pillais’ Trace = 0.10; F(6, 544) = 9.82; p < 0.001; ω = 0.10; *f* = 0.33; and
- age: Pillais’ Trace = 0.04; F(6, 544) = 3.74; p < 0.01; ω = 0.04; *f* = 0.20; but no significant interaction between strategy and pre-crisis reputation (H2): Pillais’ Trace = 0.05; F(24, 2188) = 1.26; p = 0.18; ω < 0.01; *f* = 0.12.

The follow-up analyses revealed no main effects for the strategies for the outcome variables intention (H1a) and acceptance (H1b; all p values > 0.1; ω < 0.01; *f* < 0.07). Regarding the covariates, also most effects were not significant but women and older participants had higher ratings of acceptance and intention. In contrast, the strategy manipulation affected participants’ ratings of the mayor’s benevolence (H1c; F(4, 548) = 12.28; p < 0.001; ω = 0.13; *f* = 0.40); integrity (H1d; F(4, 548) = 10.43; p < 0.001; ω = 0.11; *f* = 0.27); competence (H1e; F(4, 548) = 11.96; p < 0.001; ω = 0.12; *f* = 0.40); trust (H1f; F(4, 548) = 15.59; p < 0.001; ω = 0.15; *f* = 0.44); and their intention to vote (F(4, 548) = 9.17; p < 0.001; ω = 0.10; *f* = 0.35).

Pre-crisis reputation also showed significant effects on these variables. Follow-up tests using Tukey’s HSD test indicated that the main effect for strategy on ratings of benevolence, integrity, competence, and trust was driven by the fact that the deny strategy yielded more negative ratings than the diminish, rebuild, bolster, and no-response strategies (all p values < 0.001), which did not differ much from one another. The main effect for pre-crisis reputation was due to more positive ratings for the positive reputation conditions.

We also found the hypothesized interactions between strategy and pre-crisis reputation for the variables integrity (H2d; F(4, 548) = 3.10; p < 0.05; ω = 0.01; *f* = 0.15); and trust (H2f; F(4, 548) = 2.75; p < 0.05; ω = 0.01; *f* = 0.14). This interaction occurred due to the much larger differences between the diminish, rebuild, bolster, and no-response strategies in the negative pre-crisis reputation condition than in the positive pre-crisis reputation condition. The bolster strategy specifically evoked almost as negative a rating as the deny strategy in the negative condition, but it was perceived as much more positive than the deny strategy was in the positive condition. There were no significant interactions between strategy and pre-crisis reputation with regard to intention (H2a), acceptance (H2b), benevolence (H2c), and competence (H2e; all p values > 0.1). A post hoc power analysis showed that the design only had a power of 82% to detect the largest interaction effects and was not suitable for detecting effects smaller than this.

Of the eight planned mediation analyses (H3a−H3b) all but the two analyses contrasting the no-response condition to rebuild yielded insignificant estimates of the ACME (intention: est = − 0.09; 95% CI = − 0.16 to − 0.03; p < 0.001; acceptance: est = − 0.09; 95% CI = − 0.17 to − 0.01; p = 0.02). Participants in the bolster condition gave higher trust ratings than participants in the no-response condition, and participants who gave higher trust ratings in turn gave higher ratings of behavioral measures. Taken together, our hypothesis regarding the effects on acceptance of and adherence to behavioral measures could not be supported, while the hypotheses regarding the effect on trust in local officials were partially supported.

### Exploratory Analysis

Following our results, we also performed two additional analyses. First, to exclude the possibility that our manipulation was misunderstood by some participants, we used the participants’ perception of the communication strategy instead of the assigned experimental condition. This, however, did not alter the pattern of results. The behavioral ratings were not significantly different.

Second, we performed a hierarchical regression analysis to predict participants’ acceptance of (Table 4) and intention to perform (Table 5) the behavioral measures. Both yielded similar results—demographic variables, control convictions, personal concern regarding the COVID-19 pandemic, and trust all contributed to the prediction of the behavioral measures. By inspecting the variance accounted for in these models, we found that the level of personal concern regarding the COVID-19 pandemic was the single largest contributing variable. Personal concern accounted for 20% of the variance in acceptance—and 15% in intentions—above and beyond demographic characteristics and control convictions. Notably, while the amount of incremental variance accounted for by trust in the mayor was low (only about 4%), this amount was still significant (intention: F (1, 553) = 28.22; p < 0.001; acceptance: F (1, 553) = 15.90; p < 0.001).

**Table 4:** Results of hierarchical regression analysis predicting behavioral intentions

	(1)	(2)	(3)	(4)
(Intercept)	6.001 ***	5.692***	4.789***	4.598***
	(0.243)	(0.402)	(0.365)	(0.356)
Gender	− 0.252***	− 0.269***	− 0.157*	− 0.177**
	(0.070)	(0.070)	(0.063)	(0.061)
Age	0.004	0.005	0.003	0.001
	(0.002)	(0.002)	(0.002)	(0.002)
Education	0.038	0.034	0.021	0.018
	(0.039)	(0.039)	(0.034)	(0.034)
Internal control convictions		0.085*	0.055	0.044
		(0.042)	(0.037)	(0.036)
External control convictions		− 0.042	− 0.104***	− 0.107***
		(0.034)	(0.030)	(0.030)
Concern			0.320***	0.301***
			(0.026)	(0.026)
Trust				0.104***
				(0.018)
N	561	561	561	561
R2	0.025	0.044	0.248	0.290

Cells show coefficients and standard errors in parentheses. *p < 0.05; **p < 0.01; ***p < 0.001

**Table 5:** Results of hierarchical regression analysis predicting acceptance of protective behavioral measures

	(1)	(2)	(3)	(4)
(Intercept)	5.832 ***	5.791***	4.990***	4.797***
	(0.251)	(0.418)	(0.393)	(0.385)
Gender	− 0.271***	− 0.280***	− 0.180**	− 0.201**
	(0.072)	(0.073)	(0.067)	(0.066)
Age	0.005*	0.005*	0.004	0.002
	(0.003)	(0.003)	(0.002)	(0.002)
Education	0.076	0.073	0.062	0.059
	(0.040)	(0.040)	(0.037)	(0.036)
Internal control convictions		0.032	0.005	− 0.006
		(0.044)	(0.040)	(0.039)
External control convictions		− 0.034	− 0.089**	− 0.092**
		(0.035)	(0.033)	(0.032)
Concern			0.284***	0.265***
			(0.028)	(0.028)
Trust				0.104 ***
				(0.020)
N	561	561	561	561
R2	0.032	0.038	0.187	0.227

Cells show coefficients and standard errors in parentheses. *p < 0.05; **p < 0.01; ***p < 0.001

## Discussion

The aim of this study was to test the predictions of the SCCT in the domain of COVID-19. Overall, our hypotheses (H1a, H1b) regarding the effect of communication strategy on behavioral measures were not supported, because we found no evidence for differences between participants’ intention to adhere to and acceptance of behavioral measures. However, we did find the expected effects of crisis communication strategies on the evaluations of mayors (H1c−H1e). Similarly, we only found limited support for the buffering effect of the mayor’s pre-crisis reputation (H2) and no support for the hypothesized mediation effects (H3). Our exploratory analysis showed that trust in the mayor was related to participants’ intention to adhere to and acceptance of behavioral measures, but the single biggest predictor was the level of personal concern about the COVID-19 pandemic. In the following, we first discuss the absence of the predicted effects on the behavioral ratings, and second the effects of communication strategy on trust and the relationship between level of concern, trust, and behavioral measures. Finally, we describe some limitations of the study and provide a general outlook.

Similar to earlier experimental studies (Bilancini et al. 2020; Capraro and Barcelo 2020; Everett et al. 2020; Jordan et al. 2021), we did not find the predicted effects of message type on participants’ behavioral intentions. This lack of evidence might be due to the “minimal” interventions that were used to differentiate between different types of messages. In this study, this was particularly highlighted in the manipulation check, where many participants did not accurately choose the crisis communication strategy that the condition intended to depict. Importantly, participants seemed to be biased toward the rebuild strategy, which could be because this strategy is centered on initiating real change. Since the mayors’ statements were made in the context of introducing novel behavioral measures, it is understandable that participants also used this information when responding to the manipulation-check items. However, as our exploratory analysis indicates, the predicted effects were also absent when we used participants’ descriptions of the communication conditions instead of the intended communication conditions. While the lack of significant effects cannot, strictly speaking, be interpreted as a lack of effects, we believe that together with similar results from other studies, the results of this study indicate that variations in message wordings (Bilancini et al. 2020; Capraro and Barcelo 2020; Everett et al. 2020; Jordan et al. 2021) alone are not enough to drive substantial effects on behavioral intentions. This does not rule out that such minimal interventions have effects that are too small to be detected in the present paradigm. Given that the stimuli—posters in some of the earlier studies and newspaper articles—could potentially reach thousands of readers, even vanishingly small effects could be important. However, the effect size of different strategies on the intention to engage in protective behaviors we observed was so small (Cohens *f* = 0.06) that more than 5,165 participants need to be tested to establish these effects (alpha = 0.05, Power = 95%, number of groups = 5).

We did, however, find that information about a mayor’s pre-crisis reputation and the mayor’s crisis communication strategy affected participants’ trust in the mayor and their intention to vote for the mayor in upcoming elections. A mayor who was described as leading an effective response to COVID-19 was trusted more than a mayor whose past response was described as deficient. At the same time, communication patterns can strongly shape the perception of a mayor, such that even a mayor with a bad record on crisis management can garner some level of trust by choosing the right communication strategy. While we did not find strong effects of this in our study, research into the role of trust in politics in general (Lewis and Weigert 1985; Siegrist 2021), and the COVID-19 pandemic specifically (Coombs 2020; Siegrist et al. 2021), would suggest that this trust is a relevant resource for any following crisis in that it enables politicians to communicate more effectively. Echoing previous studies (Coombs and Holladay 1996; Coombs 2020), we found that the rebuild strategy was particularly effective when a mayor’s pre-crisis reputation was low. The bolster strategy was only effective when the pre-crisis reputation was high but not when it was low. The worst strategy with regard to trustworthiness, trust, and intention to vote was denying that a specific problem existed. However, we also found that in the context of a pandemic, diminishing one’s responsibility by scapegoating other cities was also highly effective. Yet, while this strategy might help individual mayors in the short term, it is vital to take into account the wider context and possible long-term effects on the overall societal coherence. From this perspective COVID-19 poses a real long-term threat on democratic structures by amplifying criticism of politicians, political processes, and political institutions (Flinders 2021). Any intervention that reduces the erosion of trust in these institutions might thus have indirect potential benefits for crisis communication.

In line with other researchers (Dohle et al. 2020; Harper et al. 2021; Šuriņa et al. 2021), we found that several demographic and attitudinal variables were related to participants’ acceptance of and adherence to behavioral measures. However, the magnitude of these associations in terms of the amount of variance they explained differed. Dohle and colleagues (2020) found that perceived risk of infection only accounted for about 3% to 5% of incremental variance explained in acceptance of and adherence to behavioral measures beyond that explained by demographic characteristics. Trust in politics added another 16% and 11% to the variance explained in acceptance of and adherence to behavioral measures. We found a much stronger association between concern and acceptance and adherence to behavioral measures, that is, concern explained 20% of the accounted for variance, while trust only added another 4%. Harper and colleagues (2021) did not use trust as a predictor of adherence to behavioral measures, but their reported correlation between fear of COVID-19 and adherence was similar to the association reported here between adherence and concern. To harmonize these disparate findings, it will be important to develop a shared set of measures. However, it could also be that these differences are due to the fact that the different studies were carried out at different points in time. Since the impact of trust on risk perception is generally higher in situations with low knowledge, the fact that our data from the second COVID-19 wave found a weaker impact of trust on acceptance and adherence than studies performed during the first wave, could be explained by more knowledge by the time of the second wave. Maybe a meta-analysis could try to tease apart these between-study differences.

The present study also highlights some specific problems for research that examines participants’ acceptance of and adherence to behavioral measures against COVID-19. First, many studies (for example, Capraro and Barcelo 2020; Dohle et al. 2020; Everett et al. 2020) have reported ceiling effects of the behavioral measures, because a vast majority of participants reports high levels of acceptance. Given the general importance of the COVID-19 pandemic and the fact that some of these behaviors—for example, meeting large groups of people indoors—are being criminalized, it may be necessary to use techniques that minimize the effects of social desirability, for example, randomized response techniques (Tracy and Fox 1981). Furthermore, the present and most other studies suffer from method-bias in that all used participants’ self-report as the main outcome variable.

Second, studies need to take into account the source of the message. While behavioral measures must be implemented and enforced at a local level, national government bodies are also an important source of information for citizens. In Germany—as in many other countries—the relationship between the different levels of government has shifted over time. Particularly at the beginning of the pandemic, there was some tension between the different local and national levels of government (Thielsch et al. 2021). Countries that managed the COVID-19 pandemic relatively successfully were able to allocate the decision-making processes and competencies to the optimal level (Christensen and Lægreid 2020).

Third, we asked participants to imagine a fictional scenario that involved a non-existing place, a non-existing situation, and non-existing persons. While we tried our best to model these as closely as possible to existing places, the level of personal involvement is likely to be low and the generalization of possible effects, especially on “behavioral measures,” is necessarily limited. As such we call for experimental field studies that systematically compare different conditions across cities, which often translate into the smallest administrative units for which health information is available. But we believe that fictional scenarios offer first insights into this complex domain.

## Conclusion

The COVID-19 pandemic has placed a heavy burden not only on the healthcare system but also on the public sector at large. In this context, leaders’ success in implementing behavioral measures relies on trust and effective transparent communication strategies (Christensen and Lægreid 2020; Thielsch et al. 2021). This study adds to the emerging field of studies regarding which messages are most effective (Capraro and Barcelo 2020; Everett et al. 2020). While we found that isolated statements alone do have measurable effects on people’s trust in politicians, they only minimally affect people’s acceptance of and adherence to behavioral measures.

## Footnotes

1 https://psyweb.uni-muenster.de/

2 Shops are usually closed on Sundays in Germany, but cities are allowed to declare some Sundays as open Sundays during which shops may open.

3 The complete preregistration is accessible at: https://aspredicted.org/ur6iv.pdf. Hypotheses H1f and H2f and H1g and H2g were added after the preregistration (but before starting data collection) to better differentiate between trustworthiness and trust.

4 The raw data, R-codes to replicate the analysis, and the questionnaire are available at https://doi.org/10.5281/zenodo.4889821.
